# Hydration Heat Control of Mass Concrete by Pipe Cooling Method and On-Site Monitoring-Based Influence Analysis of Temperature for a Steel Box Arch Bridge Construction

**DOI:** 10.3390/ma16072925

**Published:** 2023-04-06

**Authors:** Tan Zhang, Hua Wang, Yuejing Luo, Ye Yuan, Wensheng Wang

**Affiliations:** 1 Yulin Highway Development Center of Guangxi Zhuang Autonomous Region, Yulin 537000, China; 2 Bridge Engineering Research Institute, Guangxi Transportation Science and Technology Group Co., Ltd., Nanning 530007, China; 3 Hualan Design and Consulting Group Company Ltd., Nanning 530011, China; 4 Guangxi Polytechnic of Construction, Nanning 530007, China; 5 College of Transportation, Jilin University, Changchun 130025, China

**Keywords:** steel box arch bridge, hydration heat effect, water pipe cooling, sunshine temperature effect, closure temperature

## Abstract

The steel box arch bridge in this study will be subjected to various temperature effects from the construction to the operation stage, including the cement hydration heat effect and the sunshine temperature effect caused by an ambient temperature change. Therefore, it is very important to control the temperature effect of steel box arch bridges. In this study, the newly built Dafeng River Bridge is selected as the steel box arch bridge. This study aims to investigate the temperature effect including hydration heat and the sunshine temperature effect of the construction process of a rigid frame-tied steel box arch bridge. The manuscript presents that the heat dissipation performance of concrete decreases with the increase in the thickness of a mass concrete structure. The average maximum temperature values of layer No. 3 are about 1.3, 1.2, and 1.1 times the average maximum temperature value of layer No. 1 for the mass concrete of the cushion cap, main pier and arch abutment, respectively. The higher the molding temperature is, the higher the maximum temperature by the hydration heat effect is. With each 5 °C increase in the molding temperature, the maximum temperature at the core area increases by about 4~5 °C for the mass concrete. The pipe cooling method is conducive to the hydration heat control effect of mass concrete. Based on the monitored temperature change and displacement change, the influences of daily temperature change on the steel lattice beam and arch rib are analyzed. A temperature rise will cause the structure to have a certain camber in the longitudinal direction, and the longitudinal or transverse displacement caused by the sunshine temperature change is no less than the vertical displacement. Due to the symmetrical construction on both sides of the river, the arch rib deformation on both sides presents symmetrical synchronous changes. Based on 84 h of continuous temperature monitoring on-site, the changing trends of the arch back temperature and ambient temperature are consistent and their difference is small during 1:00~4:00 in the morning, which is determined as the appropriate closure time for the newly built Dafeng River Bridge.

## 1. Introduction

In recent decades, with the continuous development of highway and bridge construction in China, the number of bridges built has also increased with different spans and types [1]. The steel box arch bridge has been widely used at home and abroad due to its advantages such as light dead weight, large section torsional rigidity, large span capacity, small building height, economic beauty, etc. [2,3,4]. As an economically and practically important material, concrete is also widely used in infrastructure construction projects such as bridges [5,6]. In order to meet the requirements of bridge crossing capacity, mass concrete cushion caps, abutments, piers and other components are increasingly used in bridge construction. The hydration heat of cement is produced and results in surface tensile stress induced by the temperature difference, leading to temperature cracks if the allowable tensile strength is exceeded [7,8]. In addition, ambient temperature has a significant impact on the stress and alignment of a bridge structure, especially for steel box arch bridges, and temperature has a significant impact on the elevation and axis deviation of the lattice beam and arch rib [9]. Therefore, the problem of the bridge temperature effect is relatively complex, it is very important to control the temperature effect of steel box arch bridges with mass concrete, and it is necessary to conduct in-depth research.

Mass concrete structures first appeared in water conservancy and hydropower engineering, and there are a series of specifications from the temperature field theory to construction methods and temperature and crack control. With the increasing application of mass concrete in engineering, the relevant research is also more comprehensive and in-depth. Lawrence et al. [10] used numerical simulation and experimental verification methods to analyze four groups of mass concrete pouring blocks. The results showed that controlling the maximum temperature difference in mass concrete can ensure that the tensile stress of the concrete is not greater than the tensile strength of the concrete, thus achieving the purpose of controlling cracks. Freitas et al. [11] provided a hybrid finite element technical method that can be used to simulate the basic laws of temperature field changes in mass concrete structures, which can not only improve calculation efficiency, but also improve calculation accuracy. Honorio et al. [12] explored the thermodynamic response of mass concrete structures at the initial stage by numerical simulation analysis. They found that vertical displacement is not only limited by the foundation’s deformation, but that ambient temperature directly affects tensile stress, and that ambient temperature fluctuations in a day also have an impact on the structure. Pepe et al. [13] developed a numerical calculation program to simulate the hydrothermal reaction of concrete, fully taking into account the influence of the size of components, concrete strength grade and curing requirements. Cha et al. [14] proposed a thermal stress prediction method using experimental and analytical data, including deformation, elastic modulus and thermal stress experiments, and applied the prediction method to test the thermal stress of mass concrete pouring blocks. Klemczak et al. [15] studied the temperature stress distribution of mass concrete foundations with reinforcement based on theoretical and numerical simulation analysis, and proposed relevant reinforcement measures to control early temperature cracks. Singh et al. [16] used a 3D finite element to simulate the hydration heat of concrete under a forced cooling system with diffusion and dispersion characteristics, and found that there was high-temperature stress in the concrete around the cooling pipe. Tasri et al. [17,18] studied the influence of cooling pipe spacing and water temperature on the temperature stress field of mass concrete, and compared the temperature effect of three different materials of cooling pipe on mass concrete. The results showed that the cooling effect of steel pipe was the best, but the tensile stress was the largest. Han et al. [19] analyzed the original model with a thermal insulation formwork and postcooling system by the layered pouring method based on the temperature stress field of the mass concrete foundation.

A temperature change will make the structure show the deformation trend of “thermal expansion and cold contraction”. However, for statically indeterminate structures, because the free deformation is subject to boundary conditions, there will be great temperature stress in the structure, which may bring risks to the construction and use of the bridge structure [20,21,22]. In order to study the influence of the temperature effect on the various responses of suspension bridges, Xia et al. [23] established the finite element temperature field of the whole bridge and concluded that the most obvious structural response affected by solar radiation is the vertical deflection of stiffened beams through comparison. Xu et al. [24] established a finite element simulation model based on Wuhan Third Yangtze River Bridge to separate the thermal effects in the response of cable-stayed bridges, which provides a reference for further understanding the temperature field of long-span cable-stayed bridges. Wang et al. [25] took a steel truss bridge with two main trusses as the research object, taking into account the influence of solar radiation and the shielding effect between the members, and found that there were obvious positive and negative temperature differences between the two trusses, resulting in the uneven temperature field on the bridge structure. Based on the long-term measured data of temperature, solar radiation, wind speed and direction, Lei et al. [26] developed the maximum positive transverse temperature gradient prediction model, and obtained the transverse and longitudinal stress caused by temperature through calculation. The results showed that the tensile stress caused by a transverse temperature gradient is large and cannot be ignored. Fang et al. investigated the structural behavior including the axial strength and web crippling strength of cold-formed steel at elevated temperatures by using finite element models, and the proposed design equations could closely predict the structural behavior based on a reliability analysis [27,28]. Therefore, temperature change has a significant impact on the stress and deformation of a structure, and is an important factor to be considered in the design, construction and research of bridge structures. The temperature effect of bridge structures has always been one of the main research objects of scholars at home and abroad.

Most authors of the previous literature have conducted research on mass concrete at a certain location on bridges. Although a large number of temperature effect studies have been conducted, there is a lack of related applications of decision-making in actual bridge construction. Aiming at closing the above gap, this study investigates the cement hydration heat effect on the cushion cap, main pier and arch abutment and the appropriate closure timing based on the sunshine temperature effect in the construction process of the Dafeng River steel box arch bridge. For the mass concrete in the cushion cap, main pier and arch abutment, the pipe cooling method is used to control the hydration heat of cement, and the hydration heat control effect of mass concrete is evaluated based on the arrangement and monitoring results of temperature monitoring points. According to the collected temperature change and displacement change, the influences of daily temperature change on the steel lattice beam and arch rib are analyzed and identified, and the appropriate closure temperature of the arch back is determined for the newly built Dafeng River Bridge.

## 2. Project Overview

### 2.1. The Newly Built Dafeng River Bridge

**Figure 1:**
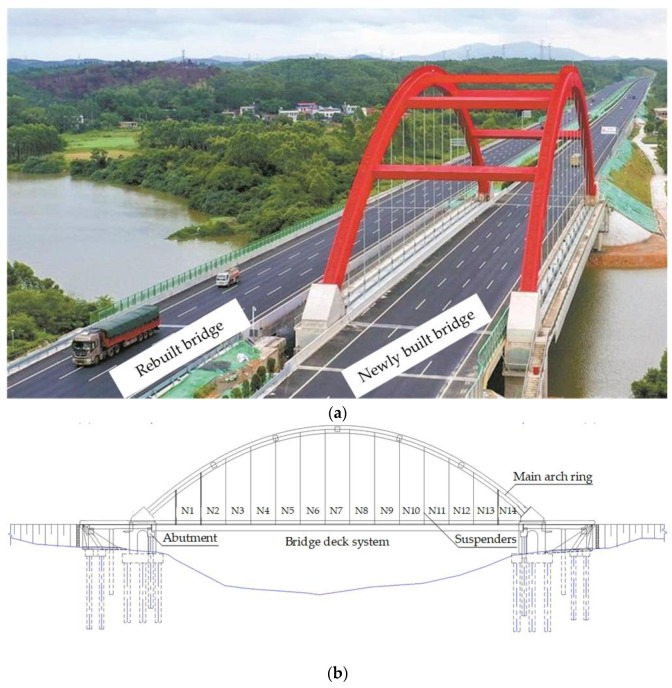
Reconstruction and expansion project of the Dafeng River Bridge in Qinzhou–Beihai section of Lanhai Expressway: (**a**) Dafeng River Bridge; (**b**) structural layout.

Dafeng River Bridge, located in Qinzhou City, Guangxi, as the main bridge in the Qinzhou–Beihai section of the Lanhai Expressway, crosses the Dafeng River at the bend of the river. The design scheme of the bridge is to demolish and rebuild the upper part and bent cap of the existing bridge without changing its existing navigation, which will be used as the left part of the whole bridge after reconstruction. The newly built Dafeng River Bridge (i.e., the right part) will be built on the downstream side. The appearance of the reconstructed whole bridge is shown in Figure 1.

The newly built Dafeng River Bridge will be a rigid frame-tied steel box arch bridge, with a clear span of 120.0 m, a clear rise of 27.0 m, and a clear rise to span ratio of 1/4.44. As shown in the elevation layout in Figure 1, the arch axis will adopt a secondary parabola, and the arch camber will be 15.0 cm. The main arch rib will be a single box and single room steel box section with an equal section, the section height will be 2.50 m, and the width will be 1.80 m. The main bridge will be a single-span main arch with two arch ribs. A total of five lateral braces will be set between the two arch ribs to ensure the overall stability of the arch bridge. The lateral brace will be a steel box structure, with a horizontal length of 24.30 m, a square section, and a height and width of 1.52 m. The thickness of the top, bottom and web of the cross brace will be 20 mm. Additionally, the suspender will adopt a whole bundle of extruded steel strands (i.e., GJ15-27 with the diameter of each steel strand being 15.20 mm and with 27 strands, Liuzhou Guiqiao Cable Co., Ltd., Liuzhou, China), with the spacing of 26.1 m in the transverse direction and 8.0 m in the longitudinal direction. There will be 14 pairs of suspenders in the whole bridge. A full anticorrosion, full-bundle, replaceable, and adjustable high-strength low-relaxation steel strand-finished cable (i.e., XGK-II 15-31 with the diameter of each steel strand being 15.20 mm and with 31 strands, Liuzhou Guiqiao Cable Co., Ltd., Liuzhou, China) will be used. Additionally, eight tie rod tensioning holes will be arranged under each arch rib, including six permanent tie rods and two reserved cable replacement holes. The bridge deck system will be a steel lattice system, which is composed of longitudinal steel beams, main beams, secondary beams and steel–concrete composite bridge decks. For the bridge deck, an 8 mm thick steel plate will be welded on the beam grid as the bottom formwork, and 15 cm thick C40 steel fiber concrete will be cast in situ. The bridge deck will be paved with 7 cm asphalt concrete.

### 2.2. Bridge Construction Based on Segmental Assembling Technique

Before construction, various preparations shall be carried out, such as site cleaning. Then, the approach bridge construction will be carried out, and the main bridge pile foundation, cushion cap, main pier and arch rib arch pedestal will be constructed at the same time. Then, the temporary construction trestle of the main bridge will be constructed. The trestle is a steel pipe pile and has a Bailey beam structure. The arch rib section shall be installed in sections to construct the main arch until the construction of the main arch rib and the transverse brace is completed and the closure of the main arch is completed. The supporting support system of the main arch will be dismantled symmetrically and orderly, and the tie rods N2, N3, N10 and N11 will be tensioned at the same time. Then, the arch rib suspenders N4~N11, and the tie rods N5, N8, N13 and N16 will be tensioned. Then, the arch rib suspenders N1–N3 and N12–N14, and tie rods N6, N7, N14 and N15 will be tensioned. Finally, the temporary trestle will be dismantled orderly and symmetrically, the tie rods N2~N8 and N10~N16 will be tensioned, and the construction of the whole bridge will be completed. The tie rods will be tensioned two times. The first tensioning control force will 1400 kN, the second tensioning control force of tie rods N2~N8 will 2200 kN, and the tensioning control force of tie rods N10~N16 will be 2250 kN. For the main material parameters of the newly built Dafeng River Bridge, Q355C will be used as an arch rib, transverse brace, and lattice beam, and Q235C will be used as a steel deck. C40, C35 and C50 will be used for the main pier, deck, sidewalk and access slab, cushion cap concrete and arch abutment concrete. *φ^s^*15.24 will be adopted as a suspender and tie rod.

### 2.3. Temperature Monitoring

The length, width and height of the cushion cap of the newly built Dafeng River Bridge will be 13.2 m, 8.4 m and 3.0 m, respectively. The length, width and height of the main pier will be 8.5 m, 4.4 m and 11.0 m, respectively, of which the length, width and height of the lower section will be 2.5 m, 4.4 m and 6.0 m, respectively, and the length, width and height of the upper section will be 8.5 m, 4.4 m and 5.0 m, respectively. The length, width and height of the arch abutment will be 7.5 m, 3.4 m and 4.923 m, respectively. Therefore, the cushion cap, main pier including the lower and upper sections, and arch abutment will be made of typical mass concrete.

**Figure 2:**
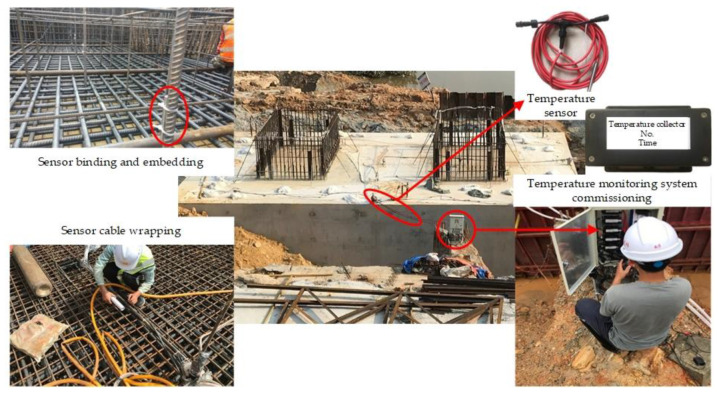
The temperature monitoring system of mass concrete in this study.

The temperature problem of the hydration heat of mass concrete is very complex, and the external temperature, construction conditions and raw material changes will cause temperature changes, which could be more accurately understood and grasped only through monitoring. During the performance of mass concrete temperature control, in order to test the construction quality and temperature control effect, the temperature control measures will be adjusted in a timely manner and improved by mastering the temperature control information. During the concrete pouring process, it will be necessary to monitor the concrete pouring temperature. During the curing process, it will also be necessary to monitor the temperature rise and fall of the concrete pouring block, the temperature difference between the inside and outside, the cooling rate and the ambient temperature. In this study, the temperature monitoring system of mass concrete is composed of a temperature sensor, data acquisition system and data transmission system. The monitoring system has the functions of displaying, storing and processing temperature and time parameters, and can draw the temperature change curve of measuring points in real-time. The number of temperature measuring points should not be less than 50. The allowable error of the temperature monitoring system shall not be greater than 0.5 °C. The test range of temperature shall be −30 °C~125 °C. Before the installation of a temperature sensor, it shall be soaked 1 m underwater for 24 h together with the transmission wire without damage. In this study, the pipe cooling method was adopted to control the thermal cracking of the massive concrete structures of the newly built rigid frame-tied steel box arch bridge. The digital temperature sensor and wireless temperature collector (shown in Figure 2) are used in a temperature monitoring system for mass concrete.

In the process of temperature measurement, the following test items need to be defined. At the same time, the layout of measuring points is closely related to that of the test items. The temperature of the concrete mixture when pouring it into the mold (i.e., the molding temperature, *T_molding_*) shall be measured at least twice per shift. The maximum temperature of concrete in the temperature measuring area is defined as the maximum temperature of concrete (*T_max_*). The concrete surface temperature (*T_surf_*) refers to the temperature at 50 mm from the concrete outer surface. The concrete bottom temperature (*T_bot_*) refers to the temperature at 50 mm from the bottom of the concrete pouring block. The difference between the maximum temperature of concrete and the surface temperature is defined as the temperature difference between the surface and interior of the concrete (Δ*T_surf_*_-*int*_). The temperature rise peak (*TRP*) is the highest temperature rise inside the concrete pouring body. The cooling rate (*CR*) refers to the temperature reduction value every day or every 4 h after the internal temperature of the concrete reaches the peak temperature under the condition of heat dissipation. The temperature value at the shady ventilation outside the structure is the concrete ambient temperature (*T_air_*).

## 3. Results and Discussion

### 3.1. Monitoring of Hydration Heat of Mass Concrete and Analysis of Early Temperature Control

#### 3.1.1. Hydration Heat of Mass Concrete in Cushion Cap

(a) Arrangement of Temperature Monitoring Points and Cooling Pipe

**Figure 3:**
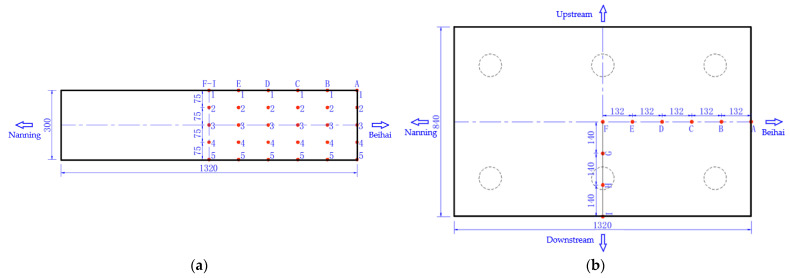
Arrangement of temperature monitoring points for mass concrete in cushion cap: (**a**) elevation; (**b**) plan (unit: cm).

During the construction process of the project, a detailed temperature monitoring scheme of hydration heat will be developed to determine a comprehensive temperature measuring point arrangement, and the hydration heat temperature of mass concrete will be monitored in real-time to guide the actual construction. Figure 3 shows the arrangement of temperature monitoring points for the mass concrete in the cushion cap. Temperature monitoring points will be arranged at the halfway point of the symmetry axis in the longitudinal and transverse directions of the cushion cap concrete, in which four measuring lines will be arranged in the transverse direction of the bridge, i.e., F, G, H and I, and six measuring lines will be arranged in the longitudinal direction, i.e., A, B, C, D, E and F. Five monitoring points will be arranged along each measuring line in the height direction. A total of 9 measuring lines (45 monitoring points in total) will be arranged for the cushion cap concrete which will be constructed first, and only 5 measuring lines (25 monitoring points in total) will be arranged for the cushion cap concrete which will be constructed later.

**Figure 4:**
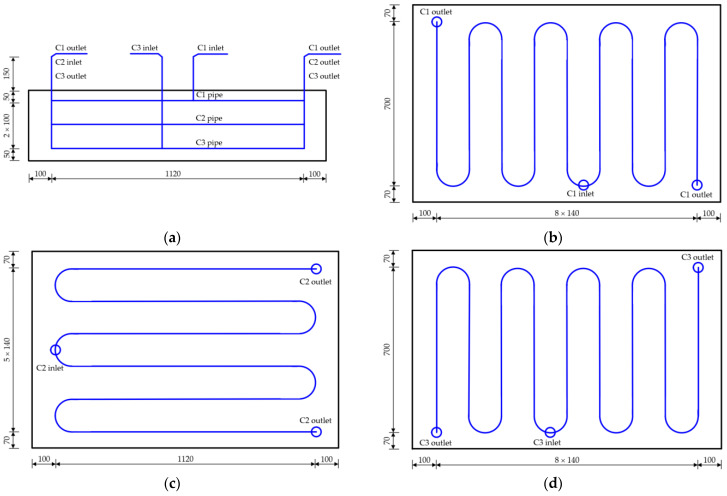
The cooling pipe layout of cushion cap: (**a**) elevation; (**b**) C1 pipe; (**c**) C2 pipe; (**d**) C3 pipe (unit: cm).

Figure 4 shows the cooling pipe layout of the cushion cap. During the construction of each cushion cap, the ambient temperature should be monitored in real-time. According to the cooling pipe layout, 1 water temperature monitoring point will be arranged at the inlet and outlet of the C1, C2 and C3 pipes, respectively, and 9 water temperature monitoring points will be arranged in total. Therefore, there will be 55 temperature monitoring points arranged for the cushion cap constructed first, of which 45 points will be temperature monitoring points of concrete, 9 points will be water temperature monitoring points at the inlet and outlet of the cooling pipe, and 1 point will be an ambient temperature monitoring point. There will be 35 temperature monitoring points arranged for the cushion cap constructed later, of which 25 points will be temperature monitoring points of concrete, 9 points will be water temperature monitoring points at the inlet and outlet of the cooling pipe, and 1 point will be the ambient temperature monitoring point.

(b) Analysis of Hydration Heat and Cracking Based on Temperature Monitoring

According to the requirements of the Chinese specification “Technical Code for Temperature Measurement and Control of Mass Concrete” (GB/T 51028-2015) [29], the molding temperature (*T_molding_*), temperature rise peak (*TRP*), the maximum temperature of concrete (*T_max_*), maximum cooling rate (*CR_max_*), the maximum temperature difference between the surface and interior (Δ*T_surf_*_-*int*-*max*_), the maximum temperature difference between the concrete surface and air (Δ*T_surf_*_-*air*-*max*_), and the maximum temperature when monitoring is stopped (*T_max_*_-*stop*_) are selected for the statistics of the temperature monitoring of a mass concrete cushion cap.

**Figure 5:**
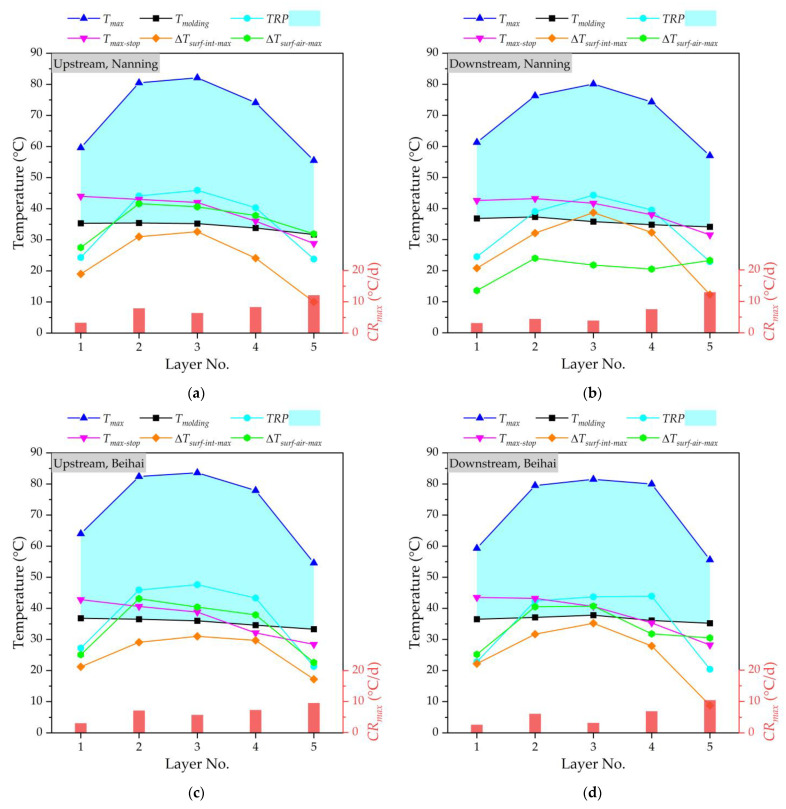
The temperature monitoring of mass concrete cushion cap: (**a**) at the upstream of Nanning side; (**b**) at the downstream of Nanning side; (**c**) at the upstream of Beihai side; (**d**) at the downstream of Beihai side.

Figure 5 shows the temperature monitoring results of a mass concrete cushion cap at the upstream and downstream of the Nanning and Beihai sides, respectively. Generally, the higher the molding temperature, the higher the maximum temperature of concrete under the action of hydration heat after pouring. It can be seen that the *T_molding_* is strictly controlled at about 35 °C in this study, and the *T_molding_* value of each layer is basically stable, in which the relatively low *T_molding_* value also avoids an accelerating hydration heat reaction. The *TRP* value is the difference between the *T_max_* and *T_molding_* values; therefore, the *TRP* and *T_max_* show a similar change trend, increasing first and then decreasing. This is because the hydration heat reaction of layered concrete is a cumulative process, and the *TRP* increases with the construction’s progress, leading to an increase in the *T_max_*. Generally, the temperature of the core area of layer No. 3 (i.e., the middle position) of layered concrete is higher than that of other layers, gradually decreasing to the outward surfaces. The heat dissipation performance of concrete decreases with the increase in the thickness of the mass concrete structure, and the layered pouring of concrete has little impact on the temperature change of the mass concrete. For the mass concrete cushion cap at four different locatitons, the average *T_max_* value of layer No. 3 is about 1.3 times the average *T_max_* value of layer No. 1. At the same time, the *TRP* value is less than 45 °C. For the pouring of mass concrete, due to the large volume of concrete, the temperature generated by the hydration heat effect in the concrete increases rapidly, while the heat dissipation is slow, resulting in a high temperature inside the concrete and a large temperature difference between the surface and the inside of the concrete, which makes it very easy for concrete cracking to occur. In this study, it could be found that the temperature difference between the surface and interior (Δ*T_surf_*_-*int*_) and the temperature difference between the concrete’s surface and air (Δ*T_surf_*_-*air*_) will be within the control range. With the help of the cooling pipe layout of the cushion cap, the cooling rate inside the mass concrete will be well controlled. The pipe cooling method is conducive to the hydration heat control effect of mass concrete, and can effectively reduce the central temperature of concrete structures. The cooling rate of mass concrete fluctuates greatly due to many influencing factors. Due to the strong heat dissipation capacity of the surface layer, the cooling rate of layer No. 5 is much higher than that of the other layers. The *T_max_* value of layer No. 5 decreases by an average of 9% compared to the lowest *T_max_* value of the other layers. When the monitoring is stopped, the maximum temperature (*T_max_*_-*stop*_) of each layer will be basically near the molding temperature (*T_molding_*). At the same time, the difference between the maximum temperature of concrete and the minimum temperature of the environment for three consecutive days can be controlled at 20 °C when the monitoring is stopped. By performing temperature control through cooling water, there will be no visible cracks in the mass concrete of the cushion cap at the upstream and downstream of the Nanning and Beihai sides.

#### 3.1.2. Hydration Heat of Mass Concrete in Main Pier

(c) Arrangement of Temperature Monitoring Points and Cooling Pipe

**Figure 6:**
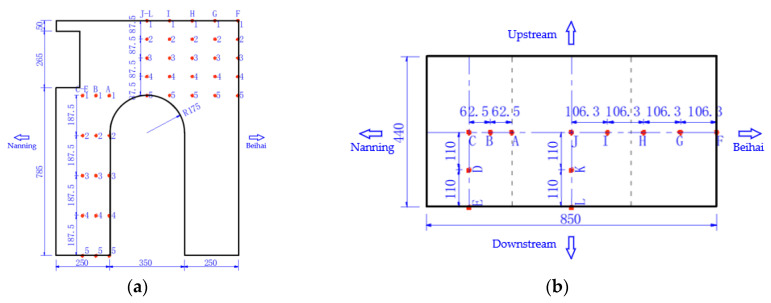
The arrangement of temperature monitoring points for mass concrete in main pier: (**a**) elevation; (**b**) plan (unit: cm).

Temperature monitoring points will be arranged at the halfway point of the symmetry axis in the longitudinal and transverse directions of the main pier concrete, as shown in Figure 6. Three measuring lines will be arranged in the transverse direction of the bridge for the lower main pier constructed first, i.e., C, D and E, and three measuring lines will be arranged in the longitudinal direction of the bridge, i.e., A, B and C. Similarly, five monitoring points will be arranged along each measuring line in the height direction. Thus, a total of five measuring lines (25 monitoring points in total) will be arranged for the concrete of the lower main pier constructed first. Only three measuring lines including A, C and E (15 monitoring points in total) will be arranged for the lower main pier concrete constructed later. On the other hand, three measuring lines will be arranged in the transverse direction of the bridge for the upper main pier constructed first, i.e., J, K and L, and five measuring lines will be arranged in the longitudinal direction of the bridge, i.e., F, G, H, I and J. Thus, a total of seven measuring lines (35 monitoring points in total) will be arranged for concrete of the upper main pier constructed first. Only four measuring lines including F, H, J and L (20 monitoring points in total) will be arranged for the upper main pier concrete constructed later.

**Figure 7:**
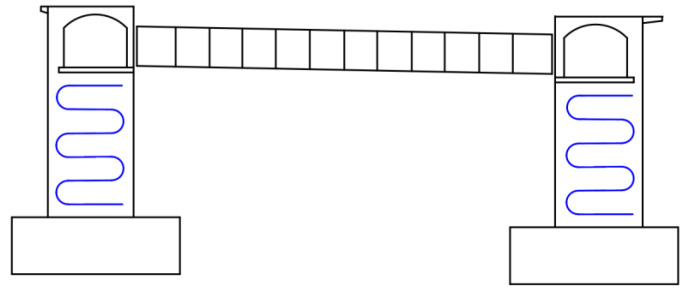
Cooling pipe layout of main pier.

Figure 7 shows the cooling pipe layout of the main pier. According to the cooling pipe layout, one temperature monitoring point will be arranged at the inlet and outlet of the pipe near the electric box for the lower main pier, two temperature monitoring points will be arranged at the cooling pipe, and eight water temperature monitoring points will be arranged at the inlet and outlet of the cooling pipe at the four main piers. In addition, one ambient temperature monitoring point will be arranged near the upper and lower main piers, two temperature monitoring points will be arranged at the pier body, and eight ambient temperature monitoring points will be arranged at the four main piers.

(d) Analysis of Hydration Heat and Cracking Based on Temperature Monitoring

**Figure 8:**
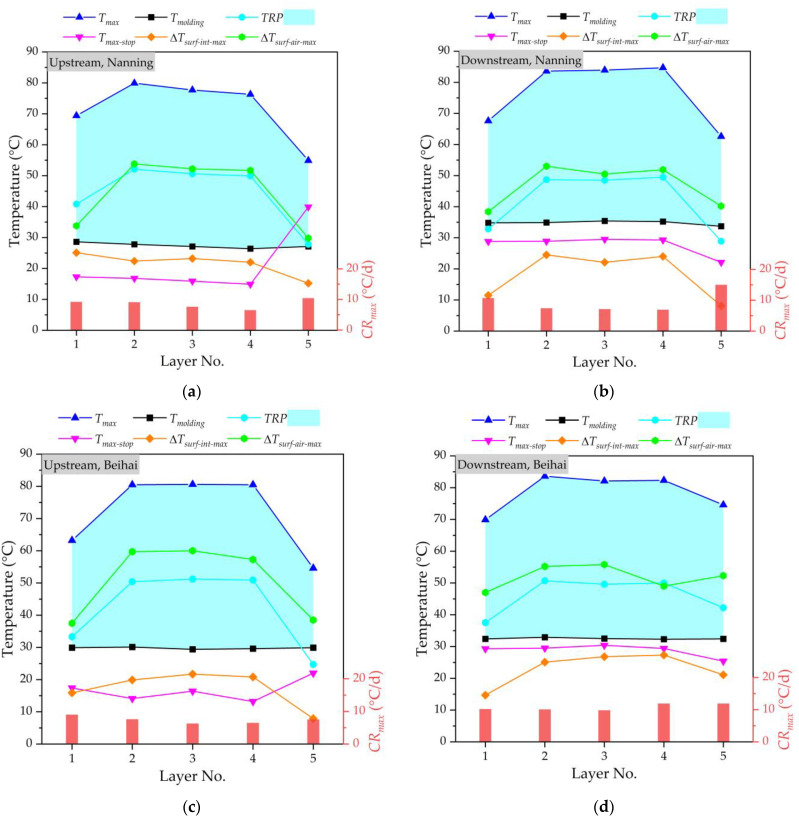
Temperature monitoring of mass concrete in lower main pier: (**a**) at the upstream of Nanning side; (**b**) at the downstream of Nanning side; (**c**) at the upstream of Beihai side; (**d**) at the downstream of Beihai side.

**Figure 9:**
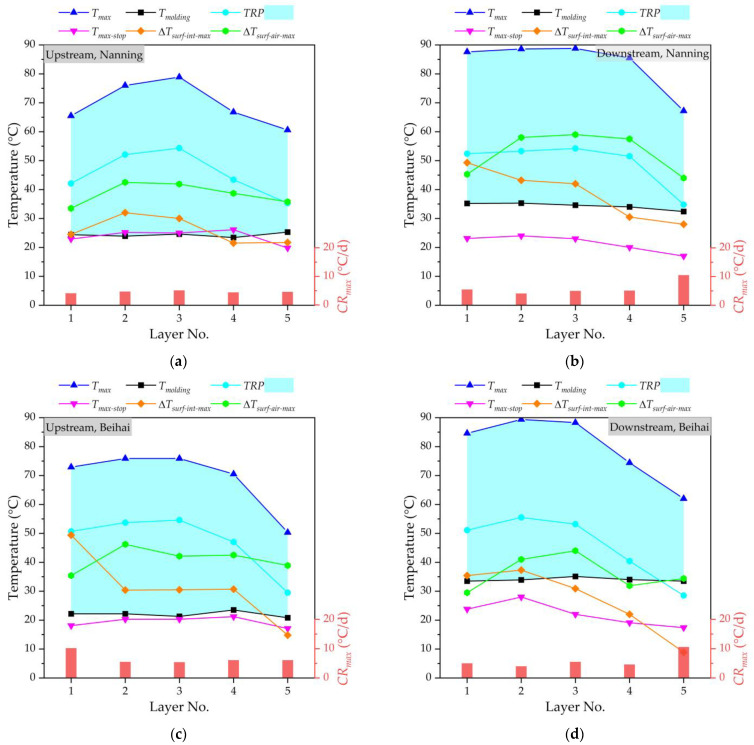
Temperature monitoring of mass concrete in the upper main pier: (**a**) at the upstream of Nanning side; (**b**) at the downstream of Nanning side; (**c**) at the upstream of Beihai side; (**d**) at the downstream of Beihai side.

Figure 8 and Figure 9 show the temperature monitoring results of the mass concrete in the lower and upper main piers at the upstream and downstream of the Nanning and Beihai sides, respectively. It can be seen that the *T_molding_* of the lower and upper main piers was strictly controlled below 35 °C in this study, and the *T_molding_* value of each layer was basically stable, in which the relatively low *T_molding_* value also avoided accelerating the action of hydration heat after pouring. According to the comparative temperature analysis of the upstream and downstream with different *T_molding_* values, it can be seen that the ambient temperature of concrete has a certain influence on its temperature field. The higher the molding temperature is, the higher the maximum temperature value by the hydration heat effect is. With each 5 °C increase in the *T_molding_* value, the *T_max_* value at the core area of layer No. 3 increased by about 4.5 °C for the mass concrete in the lower main pier, and the *T_max_* value of the core area of layer No. 3 could even be raisedx by about 5.1 °C for the mass concrete in the upper main pier. Considering the stable *T_molding_* value and *TRP* = (*T_max_* − *T_molding_*), the *TRP* and *T_max_* showed basically the same change trend, increasing first and then decreasing. This is because the hydration heat reaction of layered concrete is a cumulative process, and the *TRP* increases with the construction progress, leading to the increase in *T_max_*. The temperature of the core area at layer No. 3 (i.e., the middle position) of layered concrete was higher than that of the other layers, gradually decreasing to the outward surfaces. For the four different locations, the average *T_max_* value of layer No. 3 was about 1.2 times the average *T_max_* value of layer No. 1 for the lower main pier. Meanwhile, the maximum *TRP* value was less than 55 °C and its average value was less than 45 °C. For the pouring of mass concrete, due to the large volume of concrete, the temperature generated by the hydration heat effect in the concrete increased rapidly, while the heat dissipation was slow, resulting in a high temperature inside the concrete and a large temperature difference between the surface and the inside of the concrete, which very easily results in concrete cracking. In this study, it could be found that the temperature difference between surface and interior (Δ*T_surf_*_-*int*_) and temperature difference between concrete surface and air (Δ*T_surf_*_-*air*_) were within the control range. The average value of the Δ*T_surf_*_-*int*_ values for all layers was controlled at less than 30 °C. With the help of the cooling pipe layout of the main pier, the cooling rate inside the mass concrete was well-controlled. There were little significant increases in the *T_max_* and *TRP* values of layer No. 3, indicating that the pipe cooling method is conducive to the hydration heat control effect of mass concrete, and can effectively reduce the central temperature of concrete structures. The cooling rate of mass concrete fluctuates greatly due to many influencing factors. Due to the strong heat dissipation capacity of the surface layer, the cooling rate of layer No. 5 was much higher than that of the other layers. For the lower main pier, the *T_max_* value of layer No. 5 decreased by an average of 9% compared to the lowest *T_max_* value of the other layers. For the upper main pier, the *T_max_* value of layer No. 5 decreasesd by an average of about 19% compared to the lowest *T_max_* value of the other layers. When the monitoring is stopped, the maximum temperature (*T_max_*_-*stop*_) of each layer woill be basically lower or near the molding temperature (*T_molding_*). At the same time, the difference between the maximum temperature of concrete and the minimum temperature of the environment for three consecutive days can be controlled at 20 °C when the monitoring is stopped. By performing temperature control through cooling water, there will be no visible cracks in the mass concrete in the lower upper main piers at the upstream and downstream of Nanning and Beihai sides.

#### 3.1.3. Hydration Heat of Mass Concrete in Arch Abutment

(e) Arrangement of Temperature Monitoring Points and Cooling Pipe

**Figure 10:**
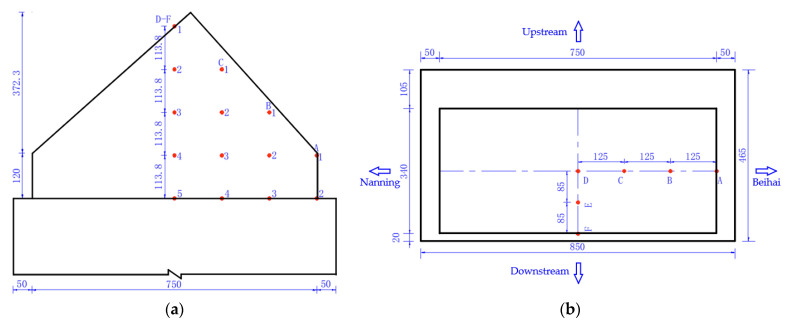
The arrangement of temperature monitoring points for mass concrete in arch abutment: (**a**) elevation; (**b**) Plan. (Unit: cm).

Temperature monitoring points will be arranged at the halfway point of the symmetry axis in the longitudinal and transverse directions of the concrete of the arch abutment constructed first, as shown in Figure 10. Three measuring lines will be arranged in the transverse direction of the bridge for the arch abutment constructed first, i.e., D, E and F, and five measuring lines will be arranged in the longitudinal direction of the bridge, i.e., A, B, C and D. Similarly, five monitoring points will be arranged along each measuring line in the height direction. Thus, a total of six measuring lines (24 monitoring points in total) will be arranged for concrete of the arch abutment constructed first. Only four measuring lines including A, C, D and F (16 monitoring points in total) will be arranged for the concrete of the arch abutment constructed later.

**Figure 11:**
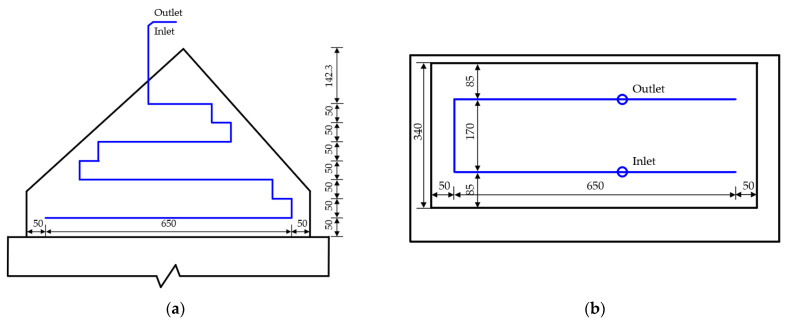
The cooling pipe layout of the arch abutment: (**a**) elevation; (**b**) plan (unit: cm).

Figure 11 shows the cooling pipe layout of the arch abutment. According to the layout of the cooling pipe, one temperature monitoring point will be arranged at the inlet and outlet of the pipe, two temperature monitoring points will be arranged at the cooling pipe of a single arch, and eight water temperature monitoring points will be arranged at the inlet and outlet of the cooling pipe for the four arch abutments.

(f) Analysis of Hydration Heat and Cracking Based on Temperature Monitoring

**Figure 12:**
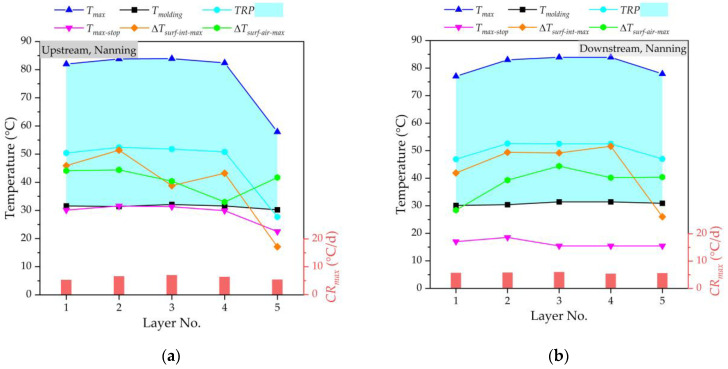

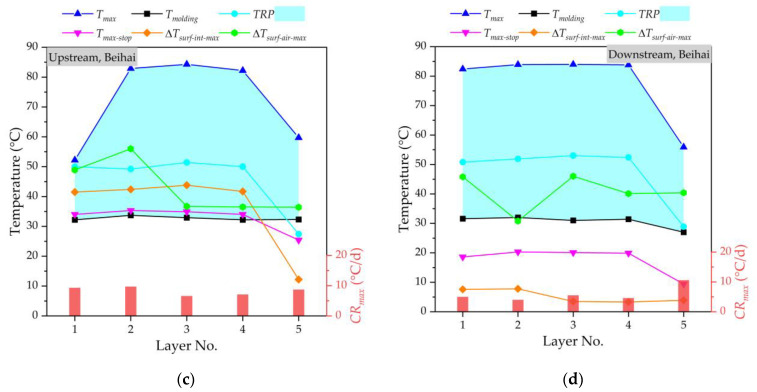
Temperature monitoring of mass concrete in arch abutment: (**a**) at the upstream of Nanning side; (**b**) at the downstream of Nanning side; (**c**) at the upstream of Beihai side; (**d**) at the downstream of Beihai side.

Figure 12 shows the temperature monitoring results of the mass concrete in the arch abutment at the upstream and downstream of the Nanning and Beihai sides, respectively. It can be seen that the *T_molding_* of the arch abutment was controlled at about 30 °C in this study, and the *T_molding_* value of each layer was basically stable, in which the relatively low *T_molding_* value also avoided accelerating the action of the hydration heat after pouring. Considering the stable *T_molding_* value and *TRP* = (*T_max_* − *T_molding_*), the *TRP* and *T_max_* showed basically the same change trend, increasing first and then decreasing. This is because the hydration heat reaction of layered concrete is a cumulative process, and the *TRP* increases with the construction’s progress, leading to the increase in the *T_max_*. Generally, the temperature of layer No. 3 (i.e., the middle position) of the layered concrete was higher than that of other layers. For the four different locations, the average *T_max_* value of layer No. 3 was about 1.1 times the average *T_max_* value of layer No. 1. At the same time, the maximum *TRP* value was less than 55 °C and its average value was less than 45 °C. For the pouring of mass concrete, due to the large volume of concrete, the temperature generated by the hydration heat effect in the concrete increased rapidly, while the heat dissipation was slow, resulting in a high temperature inside the concrete and a large temperature difference between the surface and the inside of the concrete, which very easily causes concrete cracking. In this study, it Was found that the temperature difference between the surface and interior (Δ*T_surf_*_-*int*_) and the temperature difference between the concrete’s surface and air (Δ*T_surf_*_-*air*_) were within the control range. With the help of the cooling pipe layout of the arch abutment, the cooling rate inside the mass concrete was well-controlled. The cooling rate of mass concrete fluctuated greatly due to many influencing factors. When the monitoring is stopped, the maximum temperature (*T_max_*_-*stop*_) of each layer will be basically lower or near the molding temperature (*T_molding_*). At the same time, the difference between the maximum temperature of concrete and the minimum temperature of the environment for three consecutive days can be controlled at 20 °C when the monitoring is stopped. By performing temperature control through cooling water, there will be no visible cracks in the mass concrete in the arch abutment at the upstream and downstream of the Nanning and Beihai sides.

### 3.2. Analysis of Influence Monitoring-Based Temperature Change on Bridge Deformation

#### 3.2.1. Identification of Temperature Effect on Lattice Beam Deformation

The bridge structure is located in the outdoor environment, and its internal temperature field is affected by many factors such as solar radiation, ambient temperature, etc. Due to the good thermal conductivity of steel, steel box arch bridges are more sensitive to the ambient temperature compared to ordinary reinforced concrete bridges. Ambient temperature change includes seasonal temperature change and daily temperature change. The influence of seasonal temperature changes on a structure is relatively simple, and the change is uniform. However, daily temperature change is more complex, especially under the effect of sunlight, which causes temperature differences, thus causing the displacement of the structure, and also produces the temperature’s secondary internal force for a statically indeterminate structure. The positioning of the lattice beam is the foundation of the arch rib’s assembly, and it is important to analyze the influence of temperature on the displacement of the lattice beam in construction monitoring, as it directly affects the assembly accuracy of the arch rib and the verticality of the suspender.

**Figure 13:**
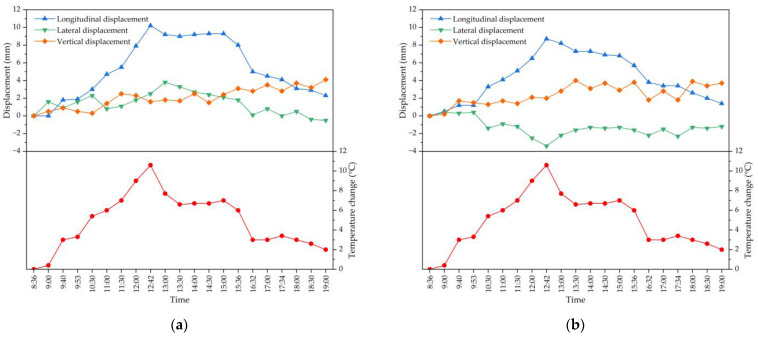
The continuous temperature and deformation monitoring of suspender N14 for the lattice beam: (**a**) on the left; (**b**) on the right.

Continuous temperature and deformation monitoring of the lattice beam was carried out to study the relationship between the position change of the suspender beam for the lattice beam and the temperature change in the outdoor environment. On the day of monitoring, it was sunny with a certain temperature difference between morning and noon. The detailed temperature–time curve relationship is shown in Figure 13. It can be seen from Figure 13 that the lattice beam will be displaced in the longitudinal, transverse and vertical directions with the change in temperature. Because the lattice beam is in the nonlinear temperature field, the structure will produce displacement, especially longitudinal displacement as the most obvious. Through the actual measurement of suspender N14 on the left and right for the lattice beam, the maximum temperature change is 10.6 °C, the maximum longitudinal displacement will be 9.3 mm, the maximum transverse displacement is 3.8 mm, and the maximum vertical displacement is 4.1 mm. From the measured displacement curve versus the outside temperature of the suspender for the lattice beam at different times, the position of monitoring points of the suspender also changed constantly with the constant change in temperature. A temperature rise will cause the structure to have a certain camber in the longitudinal direction, and the longitudinal or transverse displacement value caused by the temperature change (temperature rise or drop) under sunshine will be no less than the vertical displacement value. It is necessary to monitor the structure at a time when the temperature is relatively stable to determine the final position of the suspender; thus, the effect of temperature should be considered for the closure of the lattice beam.

#### 3.2.2. Identification of Temperature Effect on Arch Rib Deformation

**Figure 14:**
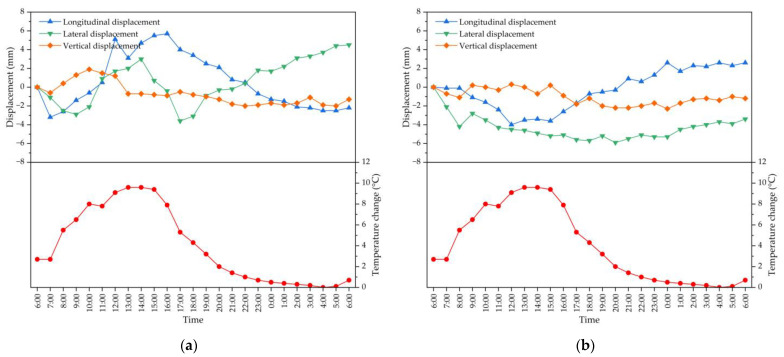
Continuous temperature and deformation monitoring of segment N4 for arch rib: (**a**) at Nanning side; (**b**) at Beihai side.

As one of the most important factors affecting the deflection of arch bridges, the ambient temperature change includes the seasonal temperature change and daily temperature change. The daily temperature change is more complex, especially under the effect of sunlight, which will cause the deflection of the arch rib, which will also lead to longitudinal, transverse and vertical deformations. The influence of seasonal temperature change on the structure is relatively simple, and its change is uniform. In order to study the relationship between the position change of segment N4 for the arch rib and temperature change in the outdoor environment, continuous temperature and deformation monitoring of the arch rib was carried out for 24 h. On the day of monitoring, it was sunny with a certain temperature difference between morning and noon, and the maximum temperature change was 9.6 °C. The detailed temperature–time curve relationship is shown in Figure 14. It can be seen from Figure 14 that the arch rib will be displaced in the longitudinal, transverse and vertical directions with the change of temperature. Because the arch rib is in the nonlinear temperature field, the structure will produce displacement, especially longitudinal and transverse displacements as the most obvious. Through the actual measurement of segment N4 on the left and right for the arch rib, the maximum longitudinal displacement is 5.7 mm, the maximum transverse displacement is 5.9 mm, and the maximum vertical displacement is 2.3 mm. From the measured displacement curve versus the outside temperature of the segment for the arch rib at different times, the position of monitoring points of the segment also changed constantly with the constant change in temperature. Additionally, due to the symmetrical construction on both sides of the river, the arch rib deformation on both sides will basically present symmetrically synchronous changes. It is necessary to monitor the structure at a time when the temperature is relatively stable to determine the final position of the segment; thus, the effect of temperature should be considered for the closure of the arch rib.

#### 3.2.3. Determination of the Appropriate Closure Timing

**Figure 15:**
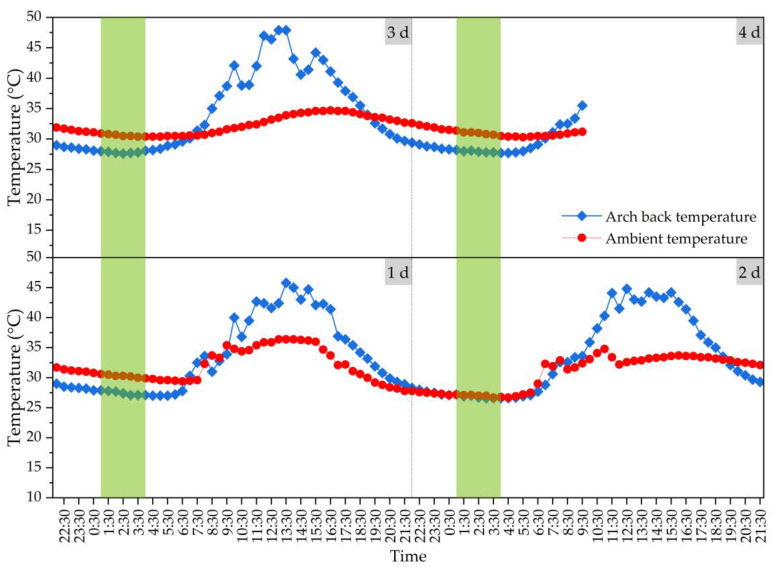
Arch back temperature at final closure and ambient temperature for 84 h (from 1 d to 4 d) before arch rib closure. (note: the green part is the appropriate closure time).

After the whole bridge is closed, the structure will become statically indeterminate, and the temperature stress generated by the bridge will be able to exceed the stress even under a live load. According to the above analysis, the structural temperature effect under the action of sunshine temperature change will be large, which will mainly have a large effect on the deflection during the bridge construction process and the stress state after the transformation of the bridge system, with a small cycle and rapid change. In order to better determine the appropriate time for arch rib closure, this study monitored the arch back temperature at the final closure and ambient temperature for 84 h before the arch rib’s closure, and the specific monitoring results are shown in Figure 15. Generally, when the temperature rises, the overall structure will rise upward, and when the temperature drops, the overall structure will deflect downward. The longitudinal displacement value of the structure due to heating or cooling will be no less than the vertical displacement value. Therefore, in the design of the arch rib’s closure, the adverse effects of heating or cooling shall be fully considered. From Figure 15, it can be seen that the minimum temperature of the arch back at the final closure before the arch rib closure occurs between 1:00~4:00 in the morning, the changing trends of the arch back temperature and ambient temperature are consistent, and the temperature difference is relatively small, so the appropriate closure time of the arch rib is determined as 1:00~4:00 in the morning.

## 4. Conclusions

This study investigates the temperature effect of the construction process of a rigid frame-tied steel box arch bridge. For the mass concrete in the cushion cap, main pier and arch abutment, the hydration heat control effect of mass concrete by the pipe cooling method is evaluated. Based on the monitored temperature change and displacement change, the influences of daily temperature change on the steel lattice beam and arch rib were analyzed and identified, and the appropriate closure temperature of the arch back was determined. From the experimental and analytical results, the following conclusions can be drawn:

(1) The heat dissipation performance of concrete decreases with the increase in the thickness of a mass concrete structure. The temperature of the core area of the middle layer was the highest for the mass concrete structures, gradually decreasing to the outward surfaces. The average *T_max_* value of layer No. 3 was about 1.3, 1.2, and 1.1 times the average *T_max_* value of layer No. 1 for the mass concrete in the cushion cap, main pier and arch abutment, respectively.
(2) The ambient temperature of concrete has a certain influence on its temperature field. The higher the molding temperature, the higher the maximum temperature value by the hydration heat effect. With each 5 °C increase in the *T_molding_* value, the *T_max_* value at the core area of layer No. 3 increased by about 4~5 °C for mass concrete. The pipe cooling method is conducive to the hydration heat control effect of mass concrete, and can effectively reduce the central temperature of concrete structures.
(3) A temperature rise will cause the structure to have a certain camber in the longitudinal direction, and the longitudinal or transverse displacement value caused by the temperature change (temperature rise or drop) under the sunshine will be no less than the vertical displacement value. Due to the symmetrical construction on both sides of the river, the arch rib deformation on both sides will basically present symmetrically synchronous changes.
(4) In view of the large structural temperature effect under the action of sunshine temperature change, the appropriate closure time of the arch rib is determined as 1:00~4:00 in the morning, during which the changing trends of arch back temperature and ambient temperature will be consistent, and the temperature difference is relatively small.

This study investigated the cement hydration heat effect of the cushion cap, main pier and arch abutment and determined the appropriate closure timing based on the sunshine temperature effect in the Dafeng River steel box arch bridge construction. However, the influence of the pipe cooling method on the hydration heat as well as the sunshine temperature effect of steel in bridge construction is worthy of further exploration.

## Data Availability

Not applicable.
